# Long-Term Survival Analysis of 5619 Total Ankle Arthroplasty and Patient Risk Factors for Failure

**DOI:** 10.3390/jcm13010179

**Published:** 2023-12-28

**Authors:** Sivakumar Allur Subramanian, Hyong Nyun Kim, SeongHyeon Kim, Jihyun Hwang, Dong I. Lee, Hye Chang Rhim, Sung Jae Kim, Lew Schon, Il-Hoon Sung

**Affiliations:** 1 Department of Orthopedic Surgery, Hallym University Dongtan Sacred Heart Hospital, Hwaseong 18450, Republic of Korea; 2 Department of Orthopedic Surgery, Hallym University Kangnam Sacred Heart Hospital, Seoul 07441, Republic of Korea; 3 Department of Biomedical Engineering, Johns Hopkins School of Medicine, Baltimore, MD 21205, USA; 4 Department of Physical Medicine and Rehabilitation, Harvard Medical School, Boston, MA 02129, USA; 5 Center for Orthopaedic Innovation, Mercy Medical Center, Baltimore, MD 21202, USA; 6 Institute for Foot and Ankle Reconstruction, Mercy Medical Center, Baltimore, MD 21202, USA; 7 Department of Orthopedic Surgery, Hanyang University Hospital, Seoul 04763, Republic of Korea

**Keywords:** total ankle arthroplasty, long-term survival, failure, risk factor

## Abstract

Background: Total ankle arthroplasty (TAA) has higher complication and failure rates compared to other surgical joint replacement procedures despite technological advances. This study aimed to find the long-term survivability of the TAA procedure and identify the patient risk factors for failure with one of the largest cohorts of patients in the literature. Methods: This retrospective cohort study involving cases between 2007 and 2018 analyzed patients who received an index primary TAA procedure in Korea. A total of 5619 cases were included in the final analysis. The TAA failure was defined as either a case with revision arthroplasty or a case with TAA implant removal and arthrodesis performed after primary TAA. Results: During the study period, the 5-year survival rate was 95.4% (95% CI, 94.7–96.1%), and the 10-year survival rate was 91.1% (95% CI, 89.1–93.1%). A younger age (<55 years, adjusted hazard ratio [AHR], 1.725; 55–64 years, AHR, 1.812; *p* < 0.001 for both), chronic pulmonary disease (AHR, 1.476; *p =* 0.013), diabetes (AHR, 1.443; *p =* 0.014), and alcohol abuse (AHR, 1.524; *p =* 0.032) showed a significantly high odds ratio for primary TAA failure in Cox regression analysis. Conclusion: The 10-year TAA survivorship rate was 91.1%. A younger age, chronic pulmonary disease, diabetes, and heavy alcohol consumption are risk factors for TAA.

## 1. Introduction

Over the past decade, several studies have been performed to identify prognostic factors for total ankle arthroplasty (TAA) [1,2,3,4,5]. Although severe mal-alignments of the ankle have been perceived as a contraindication to TAA, the development of new implant designs and evolving surgical techniques have made it possible to overcome this problem; now, even a coronal mal-alignment greater than 20 degrees can lead to good outcomes after TAA [5,6,7].

However, in spite of the recent advances in TAA technology, relatively high complication and failure rates compared to the artificial replacement of other joints still remain a major concern [8,9,10,11]. Previous studies in the literature reported that the survival rate of TAA is lower [12], and the frequency of revision procedures is higher than other joint replacement procedures [13]. The revision of the TAA procedure is troublesome for both physicians and patients due to its complexity and the diminished improvement in clinical outcomes compared to primary TAA [14].

Therefore, the need for a better understanding of factors predictive of the success or failure of TAA procedures is prevalent in the surgical field. Patient factors and comorbidities are also important factors for successful TAA, and several patient factors have been studied in the previous literature, with disagreements and no clear guidelines agreed upon. Oliver et al. analyzed 97 TAA patients and reported that obesity seems to have a negative effect on the long-term survival of TAA [3]. Meanwhile, Christopher et al. noted that TAA in obese patients seems to be safe, with 455 cases and a mean follow-up period of about 43 months [4]. The effect of diabetes on the surgical outcome of TAA has also been studied. Some works in the literature described that diabetes is an independent risk for worse outcomes after the TAA procedure [10,15,16], while another study noted that TAA may be a safe procedure in patients with diabetes [17]. Another notable study on the effect of patient factors and the surgical outcome of TAA was conducted by Daniel et al., who noted that smoking, obesity, and depression are modifiable risk factors for better implant survival in TAA with 668 patients and a mean follow-up of 1 to 2 years [18].

In a review of the previous literature, we determined that a large cohort study of TAA procedures with a long-term follow-up period is warranted for a better understanding of the effect of patient factors on TAA survival. Thus, we collected 5619 TAA cases from the national insurance data between 2007 and 2018. The purpose of the current study is to provide more evidence on which patient factors and comorbidities are related to the survival of TAA via the analysis of the long-term survivability of TAA using the largest TAA cohort among the published literature.

## 2. Materials and Methods

### 2.1. Database

This retrospective cohort study used the national population-based database of the Korean Health Insurance Review and Assessment Service (HIRA) from 2007 to 2018. In Korea, healthcare providers submit claims of patients to the HIRA to request reimbursements of medical costs from the South Korean National Health Insurance (NHI) service or medical aid programs. About 97% of Koreans are enrolled in the NHI service. Claim data submitted to HIRA includes diagnostic codes based on the International Classification of Diseases, 10th Revision (ICD-10), and all the procedures based on the Electronic Data Interchange (EDI) codes. These data also contain de-identified demographic data, including patient age, sex, hospital admissions, insurance type, and comorbidities. In addition, prescribed medication data are included in the database containing the generic name, prescription date, and duration. This study was approved by the Institutional Review Board of our institute (HDT 2020-04-012) and performed according to the tenets of the Declaration of Helsinki. The informed consent of the participants was waived because all data were totally anonymized. The Korean NHI Service provides de-identified information, and this does not affect the rights and welfare of the subject included in the database.

### 2.2. Patient Enroll Criteria and Procedures Definition

We identified all the databases, including primary TAA (EDI: N2075, N2079), revision procedure (EDI: N3715, N3719, NN4715, N4719), and implant removal (EDI: N3725, N3729, N4725, N4729) with ankle arthrodesis procedure codes (EDI: N0733, N0736) during the study period. The failure of primary TAA was defined as conversion to revision arthroplasty or ankle arthrodesis after an index primary TAA procedure. The conversion of primary TAA to revision arthroplasty was defined as a revision arthroplasty performed after the previous index primary TAA during the study period. The conversion of primary TAA to the ankle arthrodesis procedure was defined as an implant removal, simultaneously performed with the ankle arthrodesis procedure after the previous index primary TAA procedure during the study period.

**Figure 1:**
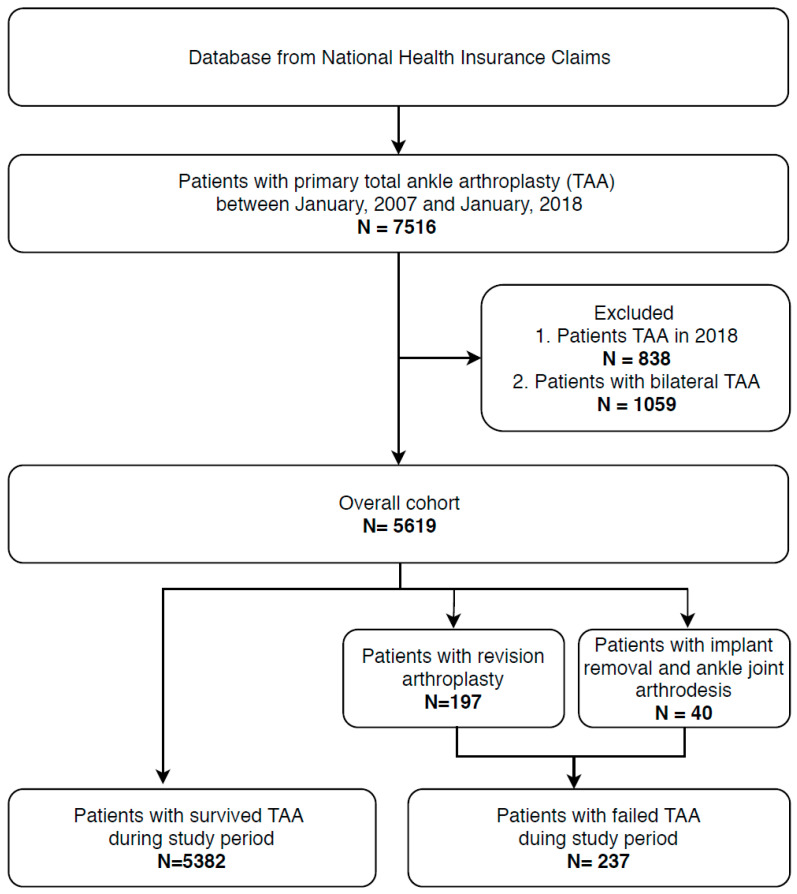
Study design.

We excluded patients with multiple primary TAA procedure codes because the current data did not provide the laterality of procedures. We also excluded patients who received the primary TAA procedure in 2018 to ensure at least one year of follow-up. The overall study design is depicted as a flow chart in Figure 1.

### 2.3. Comorbidity

Patients’ comorbidities were identified with the use of the algorithm described by Quan et al. with the ICD-10 diagnostic code [19,20,21]. Included comorbidities were a history of myocardiac infarction, congestive heart failure, cerebrovascular disease, chronic pulmonary disease, hypertension, diabetes, renal failure, rheumatoid arthritis, peripheral vascular disease, hyperlipidemia, dementia, obesity, psychosis, depression, osteoporosis, and alcohol abuse. According to the algorithm described by Quan et al., myocardiac infarction can be identified with the ICD-10 disease code with I21.x, I22.x, I25.2, and congestive heart failure can be identified with the ICD-10 code with I09.9, I11.0, I13.0, I13.2, I25.5, I42.0, I42.5-9, I43.x, I50.x, P29.0 [19,21]. Other comorbidities were identified with the same procedures.

Only the comorbidities diagnosed before the index primary TAA procedure were selected as patient variables. This study was approved by the institutional review board of our institution. Patient consent was exempted by the institutional review board because all personal information was removed or deidentified from the data.

### 2.4. Study Design and Statistical Analysis

Descriptive statistics were used for the demographic data of patients. The Kaplan–Meier survival analysis of primary TAA was conducted with the failure of the index primary TAA procedure as the endpoint. Patients were censored in survival analysis if the patients died or no failure occurred until the end of the study period. Multivariate Cox hazard regression analysis was performed to find significant risk factors for the failure of primary TAA. The hazard ratio and 95% confidence intervals (CIs) were calculated. We included age groups, sex, hospital size, admission duration for the primary TAA procedure, and comorbidities as independent variables for the regression analysis. All patients were divided into three age groups: <55, 55 to 64, and ≥65 years. Hospital size was divided into the following two grades: tertiary or general hospital grade and hospital or private clinic grade. Hospital grading in South Korea was performed with the following criteria: a “tertiary hospital” is a medical institution with a minimum of 20 specialized departments, a “general hospital” is an institution with more than 100 inpatient beds, a “hospital” is an institution with an inpatient bed quantity of between 30 and 99, and a “clinic” is an institution with less than 30 inpatient beds. Comorbidities were coded using the dichotomous scale. The significance level for all statistical analyses was set at 0.05. Data were analyzed with SAS Enterprise software (version 6.1; SAS Institute) and SPSS version 18.0 (IBM Corp., Armonk, NY, USA).

## 3. Results

### 3.1. Study Population

During the study period from 2007 to 2018, 7514 cases of primary TAA procedures in total were identified. A total of 1059 cases of bilateral procedures and 836 procedures performed in 2018 were excluded. Ultimately, a total of 5619 cases were included in the current study. During the study period, a total of 237 cases of TAA failure were identified. A total of 197 cases were salvaged with revision arthroplasty procedure, and 40 cases performed the implant removal and ankle arthrodesis procedure. The demographic data of patients are summarized in Table 1. Mean age was 64.9 ± 9.3 years, and 2833 patients were male (50.4%).

### 3.2. Kaplan–Meier Survival Analysis

**Figure 2:**
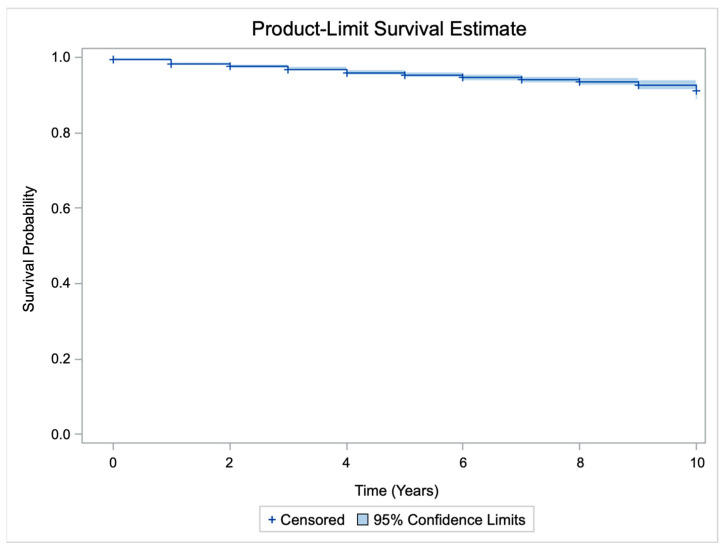
Kaplan–Meier survival curve.

The mean survival duration of primary TAA during the study period was 9.6 years (95% CI, 9.55–9.65 years). During the study period, 2.8% of patients died. The result of the Kaplan–Meier survival analysis is summarized in Table 2 and Figure 2. The 5-year survival rate was 95.4% (95% CI, 94.7–96.1%), and the 10-year survival rate was 91.1% (95% CI, 89.1–93.1%).

### 3.3. Risk Factors for Failure

Statistically significant variables identified in the multivariate Cox regression analysis are summarized in Table 3. With the reference value of patients aged over 65 years, patients aged between 55 and 64 years showed a 1.81-fold increased risk of TAA failure (adjusted hazard ratio (AHR), 1.812; *p* < 0.001), and patients aged under 55 years showed a 1.73-fold increased risk of TAA failure (AHR, 1.725; *p* < 0.001). Patients with chronic pulmonary disease showed a 1.48-fold increased risk of TAA failure (AHR, 1.476; *p* = 0.013). Diabetes showed a 1.44-fold increased risk of TAA failure (AHR, 1.443; *p* = 0.014). Alcohol abuse showed a 1.52-fold increased risk of TAA failure (AHR, 1.524; *p* = 0.032). Statistically significant variables identified in the multivariate Cox regression analysis are summarized in Table 3. With the reference value of patients aged over 65 years, patients aged between 55 and 64 years showed a 1.81-fold increased risk of TAA failure (adjusted hazard ratio (AHR), 1.812; *p* < 0.001), and patients aged under 55 years showed a 1.73-fold increased risk of TAA failure (AHR, 1.725; *p* < 0.001). Patients with chronic pulmonary disease showed a 1.48-fold increased risk of TAA failure (AHR, 1.476; *p =* 0.013). Diabetes showed a 1.44-fold increased risk of TAA failure (AHR, 1.443; *p =* 0.014). Alcohol abuse showed a 1.52-fold increased risk of TAA failure (AHR, 1.524; *p =* 0.032).

## 4. Discussion

The principal findings of this current nationwide large cohort study of primary TAA procedures, with a total of 5619 cases and 11 years of study period, included a survival rate of primary TAA at 95.4% over 5 years and 91.1% over 10 years, respectively. This is one of the largest population data related to the TAA procedure described in the literature. Risk factors associated with primary TAA failure were younger age (<55 years, AHR, 1.725; 55–64 years, AHR, 1.812), chronic pulmonary disease (AHR, 1.476), diabetes (AHR, 1.443), and alcohol abuse (AHR, 1.524).

Historically, the TAA procedure has been on a rough road. During the first ten years of its introduction to the market, less than 30% of TAA procedures showed satisfactory results [22]. Major complications included wound healing, talar collapse, and implant loosening. The fully congruous surfaces of the two-component designs were considered a cause of implant loosening, and prosthesis with a constrained design showed the highest failure rates with an up to 90% loosening rate reported during the 10 years of the follow-up period [23].

With the development of modern TAA prosthesis designs, surgical results were improved, reported survival rate varied from 85% to 96% in 5 years and 71% to 94% in more than 10 years [24]. Among the studies with a large cohort of TAA procedures, a recent study using the Swedish Ankle Registry database with 1226 cases and a 20-year study period reported a 5-year survival rate of 85% and a 10-year survival rate of 74%. They also noted that with the use of modern prostheses, survival analysis showed improved results [25]. Similarly, another study using the French exhaustive discharge database reported a 5-year survival rate of 84% and a 10-year survival rate of 78% [26].

The authors also noted that the meta-analyses using mostly non-registry data typically reported survival rates higher than the data reported by the studies using registry data only.

Despite the use of the national population-based database, the survival analysis of the current study showed relatively better patient outcomes compared to other recent studies [1,25,27]. In the literature, primary TAA showed a trend of better survival in Asian populations; a recent systematic review of TAA survival analyses in Asians with 321 TAA cases in seven studies reported 100% survival over 3 years and 95.1% survival over 5 years [24]. They also noted that the survival of TAA seemed to be better in Asian people. One study suggested that Asian patients partook in activities that promoted high ankle plantar flexion due to their lifestyle rather than heavy activities such as sports [28]. Furthermore, Valderrabano et al. noted that 56% of Western patients with TAA participated in sports activities after the procedure [29]. The difference in the lifestyles of the two populations may be the reason for the better survival of primary TAA in Asian patients, including the cohort analyzed by the current study.

Numerous studies have been conducted to identify the effect of individual patient factors on the surgical outcome of TAA procedures, including age [1,2], body mass index (BMI) [3,4], the presence of diabetes [10], and preoperative deformity [5].

The effects of age on the prognosis of primary TAA have been studied in much of the literature. Especially during the period when second-generation TAA prostheses were used, age was very significantly related to worse outcomes [30]. However, recent studies have noted that age seems to have no effect on the early outcome of primary TAA. Demetracopoulos et al. described how TAA in younger patient groups showed similar outcomes compared to older patient groups in early follow-up periods [31]. Tenenbaum et al. noted that patients in age groups between 50 and 60 years and over 70 years showed an equivalent improvement after the TAA procedure in clinical outcomes and gait patterns [32].

In the current study, patients younger than 65 years showed an increased risk of primary TAA failure. Furthermore, the group of patients younger than 55 years and the group of patients aged between 55 and 64 years showed similar adjusted hazard ratios (1.725 and 1.812, respectively). Recently, Cottom et al. reported that patients younger than 55 years showed a similar complication rate and patient satisfaction to that of patients older than 55 years [33]. However, they also found that the eldest group with patients older than 70 years had slightly better patient-reported outcomes. In addition, they noted that the younger patient groups showed a tendency for higher complication rates after TAA procedures in spite of high patient satisfaction [33]. Moreover, many studies that reported no statistically significant risk association between patient age with primary TAA failure tended to be limited in the number of patients included [31,33,34]. A recent study on a total of 4748 patients, with 677 patients under the age of 50 years, found that younger age is a significant patient risk factor, which is in agreement with the findings of our study [26].

Patients with chronic pulmonary disease showed a 1.476-fold increased risk for TAA failure in the current study. It is unclear why chronic pulmonary disease is related to TAA failure. However, chronic lung disease shares many of the same risk factors as arthritis, such as lower socioeconomic status, smoking, and obesity [35,36]. Thus, it is possible that the shared risk factors, such as smoking and obesity in chronic lung disease, negatively affect primary TAA procedures. Smoking is a common risk factor for both arthritis and chronic obstructive pulmonary disease (COPD), and it may aggravate these two disease conditions [37]. It has also been noted that smoking is associated with wound complications and lower surgical outcomes of TAA, and the recent literature recommends ceasing smoking before proceeding with TAA procedures [38,39]. A recent study also revealed that COPD is associated with a higher prevalence of joint arthritis [36]. The high prevalence of arthritis in multiple joints in patients receiving TAA procedures may alter their outcomes.

The effect of obesity on the surgical outcome of TAA has been investigated in the previous literature. Some studies found a correlation between obesity and the high failure rate of TAA [3,8], while others noted that there was no association between them [4,40]. In the current study, we could not find an association between obesity and the TAA failure rate. However, current national insurance claim data do not provide the body mass index (BMI) for each patient. Although we used a published algorithm to include comorbidities as outcome variables [19], only the patients with a diagnostic code for obesity could be identified for analysis. The lack of correlation between obesity and the risk of TAA failure in the current study should be interpreted cautiously.

Diabetes has also been reported as a risk factor for revision or infection following a TAA procedure [10,15,41]. Insulin resistance causes endothelial dysfunction, and the resulting elevated inflammatory cytokines in patients with diabetes increase susceptibility to thrombus-related complications [39]. The current study corroborates previous study outcomes; we found that the patients with diabetes showed a 1.443-fold increased risk of TAA failure.

Additionally, the excessive use of alcohol has been established as a risk factor for postoperative mortality and morbidity in various fields of medicine [42,43,44]. Ponce et al. described that patients with a preexisting alcohol use disorder have a 2.7 times greater risk of perioperative complications after shoulder joint replacement surgery [45]. Our results corroborate with the previous literature; patients with alcohol use disorder have a 1.524 times greater risk of failure after a primary TAA procedure.

Our study has some limitations inherent to database-related studies. First, we could not include radiologic analysis as an independent variable factor in the surgical results. Indeed, perioperative lower limb alignment may be an important factor for the prognosis of primary TAA. However, a large sample size with detailed data on each patient’s comorbidities and its high statistical significance can still give clinical significance to the result of the current study. Second, specific codes for each surgical procedure or diagnosis may be inaccurate. However, the accuracy of the coding system was validated by previous studies as acceptable for statistical analysis [20,46,47]. Third, the specific designs of implants used in the current study could not be identified. However, during the study period, most of the TAA implants used in Korea were the third-generation designs of the HINTEGRA^®^ ankle system (Newdeal SA, Lyon, France) and Salto Talaris Anatomic Ankle (Tornier, Saint-Ismer, France). HINTEGRA was introduced in 2004 in South Korea, and Salto Talaris in 2011. In a recent single-center study, Zafar et al. reported that there was no significant difference in the survival rates of TAA performed with the second and third-generation designs of HINTEGRA [34]. Furthermore, recent studies report comparable survival outcomes of TAA performed with third-generation designs, including HINTEGRA and Salto Talaris [26,48]. Mobility prosthesis was also used in South Korea, but it disappeared from the market in 2012. Fourth, the laterality of the procedures was not included in the database. By excluding the patients with a multiple primary TAA code, we could minimize the effect of contra-lateral side procedures.

Fifth, we could not identify combined procedures during index TAA surgery. In many cases, combined procedures like subtalar arthrodesis, hindfoot osteotomy, ligament balancing procedures, and midfoot and hindfoot correction for compensatory deformity should be performed for the optimal outcomes of TAA surgery. However, we could not include them during our data analysis session. Sixth, we could not include the etiology of procedures. The combined deformity of stage IV flatfoot or post-traumatic ankle arthritis can change the outcome of the TAA procedure. However, we believe that a large number of cases included in the current study can provide a general insight into TAA survival in South Korea. Seventh, we could not identify the severity of comorbidity. Because we used the national insurance claim database, only the accompanying disease code in insurance claims was used to identify patients’ comorbidity. The severity of diabetes, obesity, or peripheral angiopathy could not be evaluated in the current study. Therefore, caution should be exercised when interpreting the effect of the patient’s comorbidity on surgical results. Eighth, we could not include the history of smoking as a patient risk factor for failure because the database system used for the current study did not provide a smoking history.

## 5. Conclusions

In conclusion, the current study on 5619 primary TAA procedures revealed a 10-year survival rate of 91.1%. A younger age, chronic pulmonary disease, diabetes, and heavy alcohol consumption significantly affected the longevity of primary TAA. Patients with these medical comorbidities can benefit from preoperative counseling regarding the longevity of primary TAA. The effects of age after primary TAA on early patient satisfaction and long-term implant survival should be investigated as a separate entity.

## Figures and Tables

**Table 1:** Patient demography and comorbidity.

Demographic Data of Total Ankle Arthroplasty Cases	
Number of patients	5619
Age, mean (range) (SD), (%), years	64.9 (17–88) (9.3)
<55	726 (12.9)
55–64	1954 (34.8)
≥65	2939 (52.3)
Sex, (%)	
Male	2833 (50.4)
Female	2786 (49.6)
Hospital size, (%)	
Tertiary or general hospital	3076 (54.7)
Hospital or private clinic	2543 (45.3)
Admission duration, mean (SD), days	16.3 (10.3)
Comorbidities, (%)	
Myocardial infarction	426 (7.6)
Congestive heart failure	613 (10.9)
Cerebrovascular disease	1284 (22.9)
Chronic pulmonary disease	1221 (21.7)
Hypertension	3508 (62.4)
Diabetes	2408 (42.9)
Renal failure	175 (3.1)
Rheumatoid arthritis	775 (13.8)
Peripheral vascular disease	2159 (38.4)
Hyperlipidemia	3395 (60.4)
Dementia	262 (4.7)
Obesity	86 (1.5)
Psychosis	179 (3.2)
Depression	884 (15.7)
Osteoporosis	2855 (50.8)
Alcohol abuse	577 (10.3)

**Table 2:** Kaplan–Meier survival analysis.

	Survival Rate	95% Confidence Interval
1 year	98.2%	97.9% to 98.5%
3 years	96.7%	96.2% to 97.2%
5 years	95.4%	94.7% to 96.1%
10 years	91.1%	89.1% to 93.1%

**Table 3:** Results of multivariable Cox regression analysis for risk factors of failure.

	Adjusted Hazard Ratio	*p*-Value
Age (years)		
<55	1.725 (1.192 to 2.257)	*p* < 0.001
55–64	1.812 (1.355 to 2.422)	*p* < 0.001
≥65	Reference	
Chronic pulmonary disease	1.476 (1.084 to 2.010)	*p* = 0.013
Diabetes	1.443 (1.076 to 1.936)	*p* = 0.014
Alcohol abuse	1.524 (1.037 to 2.241)	*p* = 0.032

## Data Availability

The data used in the current analysis were limited to the time period that the government institute was allowed to use for scholarly purposes. Therefore, no data are available from after the current study analysis was finished.

## References

[1] Barg A., Zwicky L., Knupp M., Henninger H.B., Hintermann B. (2013). HINTEGRA Total Ankle Replacement: Survivorship Analysis in 684 Patients. J. Bone Jt. Surg. Am..

[2] Brunner S., Barg A., Knupp M., Zwicky L., Kapron A.L., Valderrabano V., Hintermann B. (2013). The Scandinavian Total Ankle Replacement: Long-Term, Eleven to Fifteen-Year, Survivorship Analysis of the Prosthesis in Seventy-Two Consecutive Patients. J. Bone Jt. Surg. Am..

[3] Schipper O.N., Denduluri S.K., Zhou Y., Haddad S.L. (2016). Effect of Obesity on Total Ankle Arthroplasty Outcomes. Foot Ankle Int..

[4] Gross C.E., Lampley A., Green C.L., DeOrio J.K., Easley M., Adams S., Nunley J.A. (2016). The Effect of Obesity on Functional Outcomes and Complications in Total Ankle Arthroplasty. Foot Ankle Int..

[5] Reddy S.C., Mann J.A., Mann R.A., Mangold D.R. (2011). Correction of Moderate to Severe Coronal Plane Deformity with the STAR Ankle Prosthesis. Foot Ankle Int..

[6] Cody E.A., Bejarano-Pineda L., Lachman J.R., Taylor M.A., Gausden E.B., DeOrio J.K., Easley M.E., Nunley J.A. (2019). Risk Factors for Failure of Total Ankle Arthroplasty with a Minimum Five Years of Follow-Up. Foot Ankle Int..

[7] Cho J., Yi Y., Ahn T.K., Choi H.J., Park C.H., Chun D.I., Lee J.S., Lee W.C. (2015). Failure to Restore Sagittal Tibiotalar Alignment in Total Ankle Arthroplasty: Its Relationship to the Axis of the Tibia and the Positioning of the Talar Component. Bone Jt. J..

[8] Sansosti L.E., Van J.C., Meyr A.J. (2018). Effect of Obesity on Total Ankle Arthroplasty: A Systematic Review of Postoperative Complications Requiring Surgical Revision. J. Foot Ankle Surg..

[9] Zaidi R., Cro S., Gurusamy K., Siva N., Macgregor A., Henricson A., Goldberg A. (2013). The Outcome of Total Ankle Replacement: A Systematic Review and Meta-Analysis. Bone Jt. J..

[10] Schipper O.N., Jiang J.J., Chen L., Koh J., Toolan B.C. (2015). Effect of Diabetes Mellitus on Perioperative Complications and Hospital Outcomes after Ankle Arthrodesis and Total Ankle Arthroplasty. Foot Ankle Int..

[11] Raikin S.M., Kane J., Ciminiello M.E. (2010). Risk Factors for Incision-Healing Complications Following Total Ankle Arthroplasty. J. Bone Jt. Surg. Am..

[12] Gougoulias N., Khanna A., Maffulli N. (2010). How Successful Are Current Ankle Replacements? A Systematic Review of the Literature. Clin. Orthop. Relat. Res..

[13] Hintermann B., Zwicky L., Knupp M., Henninger H.B., Barg A. (2013). HINTEGRA Revision Arthroplasty for Failed Total Ankle Prostheses. J. Bone Jt. Surg. Am..

[14] Lachman J.R., Ramos J.A., Adams S.B., Nunley J.A., Easley M.E., DeOrio J.K. (2019). Patient-Reported Outcomes Before and After Primary and Revision Total Ankle Arthroplasty. Foot Ankle Int..

[15] Choi W.J., Lee J.S., Lee M., Park J.H., Lee J.W. (2014). The Impact of Diabetes on the Short- to Mid-Term Outcome of Total Ankle Replacement. Bone Jt. J..

[16] Patton D., Kiewiet N., Brage M. (2015). Infected Total Ankle Arthroplasty: Risk Factors and Treatment Options. Foot Ankle Int..

[17] Gross C.E., Green C.L., DeOrio J.K., Easley M., Adams S., Nunley J.A. (2015). Impact of Diabetes on Outcome of Total Ankle Replacement. Foot Ankle Int..

[18] Cunningham D.J., DeOrio J.K., Nunley J.A., Easley M.E., Adams S.B. (2019). The Effect of Patient Characteristics on 1 to 2-Year and Minimum 5-Year Outcomes After Total Ankle Arthroplasty. J. Bone Jt. Surg. Am..

[19] Quan H., Sundararajan V., Halfon P., Fong A., Burnand B., Luthi J.C., Saunders L.D., Beck C.A., Feasby T.E., Ghali W.A. (2005). Coding Algorithms for Defining Comorbidities in ICD-9-CM and ICD-10 Administrative Data. Med. Care.

[20] Yoon J.R., Ko S.N., Jung K.Y., Lee Y., Park J.O., Shin Y.S. (2019). Risk of Revision Following Total Knee Arthroplasty or High Tibial Osteotomy: A Nationwide Propensity-Score-Matched Study. J. Bone Jt. Surg. Am..

[21] Jeschke E., Gehrke T., Gunster C., Hassenpflug J., Malzahn J., Niethard F.U., Schrader P., Zacher J., Halder A. (2016). Five-Year Survival of 20,946 Unicondylar Knee Replacements and Patient Risk Factors for Failure: An Analysis of German Insurance Data. J. Bone Jt. Surg. Am..

[22] Gougoulias N.E., Khanna A., Maffulli N. (2009). History and Evolution in Total Ankle Arthroplasty. Br. Med. Bull..

[23] Wynn A.H., Wilde A.H. (1992). Long-Term Follow-up of the Conaxial (Beck-Steffee) Total Ankle Arthroplasty. Foot Ankle.

[24] Angthong C., Chumchuen S., Khadsongkram A. (2013). A Systematic Review of Intermediate-Term Outcomes and Failure Rates for Total Ankle Replacements: An Asian Perspective. Foot Ankle Surg..

[25] Unden A., Jehpsson L., Kamrad I., Carlsson A., Henricson A., Karlsson M.K., Rosengren B.E. (2020). Better Implant Survival with Modern Ankle Prosthetic Designs: 1,226 Total Ankle Prostheses Followed for up to 20 Years in the Swedish Ankle Registry. Acta Orthop..

[26] Dagneaux L., Nogue E., Mathieu J., Demoulin D., Canovas F., Molinari N. (2022). Survivorship of 4,748 Contemporary Total Ankle Replacements from the French Discharge Records Database. J. Bone Jt. Surg..

[27] Clough T., Bodo K., Majeed H., Davenport J., Karski M. (2019). Survivorship and Long-Term Outcome of a Consecutive Series of 200 Scandinavian Total Ankle Replacement (STAR) Implants. Bone Jt. J..

[28] Lee K.T., Choi J.H., Lee Y.K., Young K.W., Kim J.B., Kim J.S., Kim W.J., Kim J.H., Lee J.Y. (2012). Functional Disabilities and Issues of Concern for Asian Patients before Total Ankle Arthroplasty. Orthopedics.

[29] Valderrabano V., Pagenstert G., Horisberger M., Knupp M., Hintermann B. (2006). Sports and Recreation Activity of Ankle Arthritis Patients before and after Total Ankle Replacement. Am. J. Sports Med..

[30] Spirt A.A., Assal M., Hansen S.T. (2004). Complications and Failure after Total Ankle Arthroplasty. J. Bone Jt. Surg. Am..

[31] Demetracopoulos C.A., Adams S.B., Queen R.M., DeOrio J.K., Nunley J.A., Easley M.E. (2015). Effect of Age on Outcomes in Total Ankle Arthroplasty. Foot Ankle Int..

[32] Tenenbaum S., Bariteau J., Coleman S., Brodsky J. (2017). Functional and Clinical Outcomes of Total Ankle Arthroplasty in Elderly Compared to Younger Patients. Foot Ankle Surg..

[33] Cottom J.M., Graney C.T., Douthett S.M., Sisovsky C., McConnell K.K., Plemmons B.S. (2020). Age-Related Outcomes in Total Ankle Arthroplasty: An Analysis of 112 Patients. J. Foot Ankle Surg..

[34] Zafar M.J., Kallemose T., Benyahia M., Ebskov L.B., Penny J.Ø. (2020). 12-Year Survival Analysis of 322 Hintegra Total Ankle Arthroplasties from an Independent Center. Acta Orthop..

[35] Mannino D.M., Buist A.S. (2007). Global Burden of COPD: Risk Factors, Prevalence, and Future Trends. Lancet.

[36] Liu Y., Wheaton A.G., Murphy L.B., Xu F., Croft J.B., Greenlund K.J. (2019). Chronic Obstructive Pulmonary Disease and Arthritis Among US Adults, 2016. Prev. Chronic Dis..

[37] Arnson Y., Shoenfeld Y., Amital H. (2010). Effects of Tobacco Smoke on Immunity, Inflammation and Autoimmunity. J. Autoimmun..

[38] Lampley A., Gross C.E., Green C.L., DeOrio J.K., Easley M., Adams S., Nunley J.A. (2016). Association of Cigarette Use and Complication Rates and Outcomes Following Total Ankle Arthroplasty. Foot Ankle Int..

[39] Cody E.A., Scott D.J., Easley M.E. (2018). Total Ankle Arthroplasty: A Critical Analysis Review. J. Bone Jt. Surg. Rev..

[40] Bouchard M., Amin A., Pinsker E., Khan R., Deda E., Daniels T.R. (2015). The Impact of Obesity on the Outcome of Total Ankle Replacement. J. Bone Jt. Surg. Am..

[41] Escudero M.I., Le V., Barahona M., Symes M., Wing K., Younger A., Veljkovic A., Penner M. (2019). Total Ankle Arthroplasty Survival and Risk Factors for Failure. Foot Ankle Int..

[42] Tonnesen H., Nielsen P.R., Lauritzen J.B., Moller A.M. (2009). Smoking and Alcohol Intervention before Surgery: Evidence for Best Practice. Br. J. Anaesth..

[43] Rotevatn T.A., Boggild H., Olesen C.R., Torp-Pedersen C., Mortensen R.N., Jensen P.F., Overgaard C. (2017). Alcohol Consumption and the Risk of Postoperative Mortality and Morbidity after Primary Hip or Knee Arthroplasty—A Register-Based Cohort Study. PLoS ONE.

[44] de Wit M., Goldberg A., Chelmow D. (2013). Alcohol Use Disorders and Hospital-Acquired Infections in Women Undergoing Cesarean Delivery. Obstet. Gynecol..

[45] Ponce B.A., Oladeji L.O., Raley J.A., Menendez M.E. (2015). Analysis of Perioperative Morbidity and Mortality in Shoulder Arthroplasty Patients with Preexisting Alcohol Use Disorders. J. Shoulder Elb. Surg..

[46] Kimm H., Yun J.E., Lee S.H., Jang Y., Jee S.H. (2012). Validity of the Diagnosis of Acute Myocardial Infarction in Korean National Medical Health Insurance Claims Data: The Korean Heart Study (1). Korean Circ. J..

[47] Cho S.K., Sung Y.K., Choi C.B., Kwon J.M., Lee E.K., Bae S.C. (2013). Development of an Algorithm for Identifying Rheumatoid Arthritis in the Korean National Health Insurance Claims Database. Rheumatol. Int..

[48] Zhao D., Huang D., Zhang G., Wang X., Zhang T., Ma X. (2020). Positive and Negative Factors for the Treatment Outcomes Following Total Ankle Arthroplasty? A Systematic Review. Foot Ankle Surg..

